# Functionality of cooperation between health, welfare and education sectors serving children and families

**DOI:** 10.5334/ijic.1070

**Published:** 2013-12-02

**Authors:** Outi Kanste, Nina Halme, Marja-Leena Perälä

**Affiliations:** National Institute for Health and Welfare, Oulu, Finland; National Institute for Health and Welfare, Helsinki, Finland; National Institute for Health and Welfare, Helsinki, Finland

**Keywords:** child, cooperation, family, integration, municipality

## Abstract

**Introduction:**

Children and their families use a lot of different services, which poses challenges in terms of cooperation between service providers. The purpose of the study was to evaluate and compare the functioning of this cooperation between services for children and families in Finland's mainland municipalities from the viewpoints of employees and managers.

**Method:**

The study was carried out using a cross-sectional survey design. Data were gathered using two postal surveys from employees and managers working in health care, social welfare and educational settings. The data consisted of responses from 457 employees and 327 managers.

**Results:**

Employees working in primary health care and education services assessed cooperation as working better than did those working in social welfare, special health care or mental health and substance abuse services. Well-functioning cooperation at the operational and strategic level was related to good awareness of services and to agreed and well-functioning cooperation practices with few barriers to cooperation. Employees were more critical than managers concerning the occurrence of barriers and about the agreed cooperation practices.

**Conclusions:**

Successful cooperation in providing services for children and families requires an awareness of services, management structures that support cooperation, agreed practices and efforts to overcome barriers to cooperation.

## Introduction

Children and their families represent a significant population group that use a lot of different services in health care, social welfare, education and other sectors, either at the same time or at different stages of the lifecycle [1–3]. Despite the fact that today a significant proportion of children and families are doing well, many still have multiple needs for support, assistance and services: for example, people with disabilities, those at risk of abuse and chronically ill children as well as families with multiple problems or substance abuse and mental health problems have complex needs and require multiple support. This presents challenges for service providers to cooperate in meeting the needs of children and families in an appropriate, timely and customer-oriented way.

Although the strengthening of cooperation and the development of cooperation structures between service providers have been a key issue in health, social and education policy in many Western countries [3–8], some children and families feel that services are nevertheless fragmented and that cooperation between service providers and different sectors is not functioning optimally. The fragmentation and differentiation of the service system together with insufficient cooperation may reduce access for children and families to services as well as influencing some to drop out of the service system [9,10]. Families with multiple needs or substance abuse and mental health problems have difficulties in accessing services which best meet their needs, for example, in primary and special health care, social welfare and mental health and substance abuse services.

The study was conducted in Finnish municipalities. Municipalities have extensive duties and obligations to provide health, social and other welfare and education and cultural services. The major part of these services is run and funded publicly by the municipalities that provide services either by themselves, jointly with other municipalities, or purchase services from other service providers such as voluntary and private organisations [11–14]. The Finnish public service system is strongly decentralised compared to other organisation for economic co-operation and development countries, while national steering is rather weak [12,14]. Since each municipality determines its own scope of coverage within general limits set by national legislation and since the extent of coordination across municipalities is limited, a fair amount of variation exists regionally in public services [11,12]. The municipal health care system has separate organisational structures in place for primary and secondary services, which have clearly hindered cooperation between these levels [11]. The schools also have the right to provide educational services according to their own administrative arrangements, as long as the basic functions, determined by law, are carried out [13].

In the last 10 years, several local reforms have been enacted to enhance cooperation between primary and secondary health care and social welfare services by integrating organisations [11]. The Finnish government's key strategy has been to create larger municipalities or enhanced cooperation among municipalities. In 2011, there were 336 municipalities in Finland, but the median population is only around 6000, which is low given the wide responsibilities devolved to municipalities, notably in the health and education sectors [14].

Children and family services in municipalities are organised in slightly different ways and are provided through a number of service providers in the public, private and voluntary sectors. A number of laws, regulations and recommendations at the national level concerning health care, social welfare and education services guide cooperation among service providers, authorities and employees. The ongoing extensive social welfare and health care service structure reforms and local government reforms are aimed at modifying the structure and integration of services for children and families [11,12]. Although regulations at national level clearly specify a strengthening of multi-disciplinary, cross-sectoral and integrated services, the recommendations and obligations are often service-specific and provide few practical guidelines for implementing cooperation [5,9].

### Functionality of cooperation in children and family services

Cooperation in children and family services can be viewed from the conceptual framework of integration in public health that was developed by Axelsson and Axelsson [15]. With increasing functional (e.g. specialisation and professionalisation) and structural differentiation of organisations involved in the pursuit of services for children and families, there is a growing need for interorganisational integration. Since most of the organisations involved are not market-oriented and many of them are not part of a common hierarchy, integration of these organisations is primarily a question of cooperation and collaboration between different organisations, but there are also elements of coordination [15].

There are many diverse and sometimes even contradictory definitions of these forms of integration. In order to sort out the relationships between concepts, a distinction can be made between vertical and horizontal integration. Vertical integration occurs between organisations or organisational units on different levels of a hierarchical structure, while horizontal integration occurs between organisations or units that are on the same hierarchical level or have the same status. Cooperation can be defined as a form of integration with a high degree of both vertical and horizontal integration. It is usually based on hierarchical management, although it can be combined with voluntary agreements and informal contacts and communications between the separate organisations. Collaboration has a high degree of horizontal integration but a low degree of vertical integration. This means that most integration is accomplished through voluntary agreements and mutual adjustments between the organisations involved. This form of integration is based on a willingness to work together and it may be implemented through intensive contacts and communications. Coordination has a high degree of vertical integration but a low degree of horizontal integration. This means that integration is achieved mainly through a common management hierarchy [15].

The literature related to concepts and theory formulation around cooperation and collaboration has been diverse [5,8,16,17]. For example, Horwath and Morrison [2] define cooperation as one level of multiagency collaboration. Cooperation can also be viewed from different organisational levels: from an operational or patient-work level and from a strategic level [5,18,19]. Inter-professional collaboration can be perceived as internal collaboration between professionals from the same organisation and external collaboration, as found between professionals from different organisations or services [8]. Willumsen [5] has stated that interprofessional collaboration can be considered between professionals and between professionals and service users on an interpersonal level, as well as between organisations or services on an interorganisational level.

In the literature on children and family services, a number of functional and structural factors related to cooperation and collaboration have been described. Problems for collaboration and cooperation can stem from the absence of a collaboration culture [20], different professional and organisational cultures, values and interests [8,15], mistrust caused by incomplete understanding of roles and responsibilities and lack of trust [2,3,6,18], insufficient knowledge of each other's activities or available services [9,20,21], inadequate feedback [3,6], differences in commitment [3,15,18] and inequality [3]. Moreover, cooperation and collaboration can be further complicated by conflicting and defensive interprofessional relationships [1], communication problems [2,18], territorial thinking and unrealistic expectations [3,6], as well as a lack of resources or an agreement on joint objectives [1,3,18,22]. Structural factors are related to the existence of separate administrative boundaries, different laws, rules and regulations, budgets, information systems and databases [9], inflexible organisational structures [2] and the absence of structures supporting collaboration and good collaboration practices [20].

In this study, cooperation in providing services for children and families is viewed from the conceptual framework of integration [15] and as internal cooperation between professionals from the same organisation and external cooperation between professionals from different organisations or services [8]. This is appropriate because several reforms in Finnish municipalities have been enacted to enhance cooperation by integrating organisation and services [11]. The functionality of cooperation is evaluated from the operational and strategic levels. This is a valuable addition to the research literature, as there are few comparative data available on those two levels from the viewpoints of employees and managers as well as concerning individual and environmental factors related to cooperation. In addition, a broad range of services in health care, social welfare and educational settings will be evaluated [5,18].

### Study purpose and research questions

The purpose of the study was to evaluate and compare the functionality of cooperation from the viewpoints of employees and managers in Finnish municipalities while considering both operational and strategic aspects. Cooperation was evaluated in primary health care, social welfare, education, special health care services, as well as in mental health and substance abuse services provided to children and families. The research questions were as follows:

- How do the employee and manager evaluate the functionality of cooperation and how do background factors (age, education, managerial position, sector, location of workplace and size of population in the municipality) relate to these evaluations?
- How do perceptions of the functionality of cooperation relate to the awareness of services, structures supporting cooperation, agreed cooperation practices and barriers to cooperation?
- How do employees’ and managers’ perceptions of the agreed cooperation practices and barriers to cooperation differ from each other?

## Methods

### Data collection

The study was carried out as a cross-sectional survey design. Data were gathered using two postal surveys in 2009 sent to employees and managers working in health care, social welfare and educational settings. Data were collected from all municipalities in the Finnish mainland, with responses received from 457 employees and 327 managers.

The employee study population was formed from operational units providing services for children and families in Finnish municipalities (*n* = 332), with five types of units included: maternity and child welfare, school health care, day care, preschool and basic education units. In those municipalities that had more than 4000 inhabitants (*n* = 209), the survey was sent to all (*n* = 1045) operational units. In addition, in those municipalities with under 4000 inhabitants (*n* = 123), a random selection of 35 maternity and child welfare, 35 school health care, 35 day care, 35 preschool and 35 basic education operational units were sent a survey. Hence, a total of 1220 questionnaires were sent to the participating units. After one survey reminder, 457 respondents returned fully completed questionnaires, giving a response rate of 37%.

The manager study population was formed from three service sectors (health care, social welfare and education) in all Finnish municipalities (*n* = 332). The data on managers were collected from the heads of these three sectors, with a total of 996 questionnaires sent to municipalities. After one reminder, the final data consisted of 327 managers’ responses that were fully completed, giving a response rate of 33%.

The study is part of a larger research project where the appropriate sample sizes have been calculated with power analysis [9,10]. In this study, power analysis was used to confirm the adequacy of data set sizes. A significance level of 95% (*α* = 0.05) and a statistical power of 80% were used. The power analysis was performed using the General Power Analysis (G*Power 3.1) software and it showed that the data sets were adequate in relation to the methods of analysis used [23,24].

The questionnaires were accompanied by a covering letter that explained briefly the purpose of the research project, emphasised that participation was voluntary and guaranteed absolute confidentiality. A completed and returned questionnaire was interpreted as an indication of consent to the research. Ethical approval (§43/2009) was obtained from the ethical committee of the National Institute of Health and Welfare.

### Participants

A total of 457 employees and 327 managers participated in the study. The participants’ characteristics are described in Tables 1 and 2.

#### Employees

Nearly half of employees were older than 50 years of age. The job titles for their work were varied, such as nurses, public health nurses, school nurses, day care nurses, kindergarten teachers and pre-school and school teachers. A little more than a third (37%) worked in front-line managerial positions. Front-line managers were examined as part of the group of employees as they are responsible for the daily management of line employees who offer the services and with whom most employees interact with on a daily basis. In contrast, the manager study population consisted of top-level managers who do not deal directly with day-to-day activities, but rather set goals for the organisation at a strategic level and direct the organisation in achieving them. Nearly all (93%) were permanent employees. About one-third worked in health care and one-third in education services (Table 1).

#### Managers

About two-thirds of the managers were older than 50 years of age and had at least a higher university degree. They held many different job titles, such as nursing directors, chief medical officers, senior physicians, leading social workers, family service or child protection managers, school principals and directors of education, day care or social services. More than a third of managers worked in a combined social welfare and health care sector and another third in education services. Managers were older and more educated than employees (Table 2).

### Measures and data analysis

The questionnaires were developed for the purposes of the study and were based on theory, previous research and multi-disciplinary knowledge of services for children and families [9,10]. Questionnaires included background factors (Tables 1 and 2). The main study variables are described in Table 3.

Employees’ perceptions of cooperation with health care, social welfare and education services were measured with 32 items on a five-point scale ranging from 1 to 5 (very good to very poor). The employees evaluated the functionality of cooperation with those employees and services in which they had been cooperating within the previous 12 months. Managers’ perceptions of cooperation with different services were measured with five items on a five-point scale ranging from 1 to 5 (very good to very poor). The managers evaluated the functionality of cooperation with those employees and services in which the cooperation was implemented within the previous 12 months. Services were formed into five subscales: (1) primary health care (e.g. maternity and child health clinics or school health nurse or doctor, dental care, psychologist, physician, nurse or public health nurse in health centre, physiotherapy, occupational, speech and nutritional therapy), (2) social welfare (e.g. education and family counselling, home help services, child protection, family worker, social worker, disability services), (3) education (e.g. pre-school and school teacher, day care, student welfare group, school social worker), (4) special health care (e.g. outpatient clinics and wards) and (5) mental health and substance abuse services (e.g. mental health outpatient services).

Awareness of services for children and families was measured with 14 items from employees and with 8 items from managers, using a five-point response scale ranging from 1 to 5 (very good to very poor). Information on the agreed cooperation practices were elicited from employees and managers using a 30-item measure consisting of six statements, which evaluated written agreements on shared goals, written agreements on joint practices, commitments to common goals, formation of joint services, information flows and written agreements on joint monitoring and evaluation. Cooperation was evaluated with a five-point scale ranging from 1 to 5 (strongly disagree to strongly agree) for each statement within sectors, between sectors, between municipalities and with third sector actors and private sector providers. Items were formed into six subscales according to the statements.

Barriers to cooperation were measured from employees’ and managers’ viewpoints by using a 19-item measure with a five-point scale (1 = very little; 5 = very much). Items were formed into four subscales: (1) work culture and attitudes (diversity of professional cultures, competition, lack of mutual trust, shortcomings in cooperation skills, commitment and lack of knowledge of other's work), (2) management practices (lack of leadership, unclear decision-making practices, different opinions of resource allocation, unclear responsibilities, inadequate flow of information, lack of common goals and cooperation structures), (3) environmental factors (physical distance, employee turnover, privacy policy) and (4) lack of resources (inadequate time, lack of financing, incomplete statistical recording).

Managers used dichotomous scales (yes/no) to evaluate the structures that support cooperation within sectors, between sectors in the municipality and between municipalities. Three subscales were formed, each consisting of 19 items measuring cooperation structures, such as resources allocated to cooperation, information systems, development projects, management teams, multi-disciplinary work groups, training, naming persons responsible for cross-administrative work, customer-oriented service coordination and written agreements on duties, responsibilities and information flow.

In the reliability analysis, Cronbach's alpha was used to measure the internal consistency of the measures. Alpha values would preferably range between 0.70 and 0.90 [25] even if values as low as 0.50 may be acceptable [26]. The internal consistencies of the subscales were satisfactory, since the alphas met the criterion of 0.50 (Table 3).

Descriptive statistics (frequency and percentage distribution, mean, standard deviation) were used to characterise the participants. To investigate the relationships between study variables and in group comparisons, we used Chi-square test, the Fisher's exact test, independent-samples *t*-test and one-way analysis of variance. The post hoc multiple comparisons were examined with Scheffe's test. The statistical analyses were made with the SPSS 19.0 software package [27] and the level of statistical significance was fixed at *p* < 0.05.

## Results

### 

#### Functionality of cooperation

Almost three-quarters (70%) of employees thought that cooperation worked fairly or very well for employees working in primary health care. The corresponding proportion for education services was 79%, for social welfare 52%, for special health care 58% and for mental health and substance abuse services 51%.

About 82% of the managers thought that cooperation with primary health care worked fairly or very well. The corresponding proportion for education services was 81%, social welfare 78%, special health care 51% and mental health and substance abuse services 50%.

Some differences in the associations of participants’ background factors and perceptions of the functionality of cooperation were statistically significant, but the differences varied slightly across service sectors, as shown in Tables 1 and 2. Employees under 40 years of age thought the cooperation with employees working in social welfare and education services functioned more poorly than did older employees. The proportion of employees aged more than 50 years was higher in health care than in other sectors [*χ*2(6) = 29.1, *p* < 0.001]. Highly educated employees thought the cooperation with employees working in primary health care functioned more poorly than did less-educated employees, though they thought the cooperation with employees working in education services worked better than did less-educated employees. The proportion of employees with at least a higher university degree was greater in education services than in other sectors [*χ*2(6) = 125.4, *p* < 0.001].

Employees working in a managerial position at the operational level thought the cooperation with employees working in primary and special health care functioned more poorly than workers who did not work in a managerial position. Managers under 40 years of age thought the cooperation functioned more poorly with employees working in special health care than older managers. Highly educated managers thought the cooperation functioned more poorly with employees working in primary and special health care than did less-educated managers.

Both employees and managers working in any sector thought the cooperation functioned best with employees working in their own service sector as opposed to with employees of other sectors. Employees thought the cooperation functioned better with employees working in social welfare in rural areas than in urban areas. On the other hand, managers thought that cooperation with employees working in social welfare and education services in municipalities with under 4000 inhabitants functioned better than in larger ones.

#### Awareness of services

Nearly one fifth (18%) of employees thought their awareness of services for children and families to be rather poor or poor. The corresponding percentage among managers was 6%. Employees’ awareness of services was associated with cooperation in all sectors: good awareness of services was related to perceptions of good cooperation (Table 4). Respectively, the managers’ good awareness of services was associated with perceptions of good cooperation in social welfare and education services as well as in mental health and substance abuse services (Table 5). There was no difference in the awareness of services according to employees’ or managers’ background factors.

#### Structures supporting cooperation

As expected, managers thought that the structures supporting cooperation in providing services to children and families were best realised within sectors and worst realised between municipalities. There were more structures in municipalities with more than 15 000 inhabitants supporting cooperation within the sector than in municipalities with under 4000 inhabitants [*χ*2(2) = 22.0, *p* < 0.001]. The structures supporting cooperation between sectors in municipalities and between municipalities were associated to some extent with well-functioning cooperation (Table 5). The evaluation of highly educated managers was that there were more structures supporting cooperation between sectors in municipalities [*χ*2(2) = 7.4, *p*=0.025] than was assessed by less-educated managers.

#### Agreed cooperation practices

Commitment to common goals was for both employees and managers the most frequently agreed cooperation practice. The second most common practice in both groups was the formation of joint services. Employees’ and managers’ perceptions differed from each other regarding the prevalence of the flow of information. Managers more than employees thought the information flow were better (Table 6).

Agreed cooperation practices were associated with employees’ perceptions of cooperation, particularly in education and social welfare services and primary health care. All six measured working practices that were assessed to be working very well were related to perceptions of good cooperation. Particularly, an assessment of working very well in regard to information flow, commitment to common goals and formation of joint services were associated with well-functioning cooperation in nearly all sectors (Table 3). According to employees in municipalities with under 4000 inhabitants, the flow of information worked better than in municipalities with more than 15,000 inhabitants [*χ*2(2) = 6.4, *p* = 0.042]. In rural areas, the commitment to common goals [*χ*2(1) = 5.1, *p* = 0.026] and agreement on monitoring and evaluation [*χ*2(1) = 4.2, *p* = 0.042] were evaluated to be more significant than in urban areas. The educational level of employees was related to some agreed cooperation practices. Highly educated employees more than less-educated employees thought that the agreement on joint practices [*χ*2(2) = 9.5, *p* = 0.009], flow of information [*χ*2(2) = 8.5, *p* = 0.015] as well as agreement on monitoring and evaluation [*χ*2(2) = 10.9, *p* = 0.004] worked better.

Managers’ perceptions of agreed cooperation practices were associated with perceptions of cooperation, particularly in education services. All six measured working practices that were assessed to be working very well were related to well-functioning cooperation. Agreed cooperation practices and particularly flow of information were related to good cooperation in all sectors (Table 4). In municipalities with under 4000 inhabitants, the formation of joint services worked better than in municipalities with more than 15,000 inhabitants [*χ*2(2) = 6.6, *p* = 0.036]. Highly educated managers were more likely than less-educated managers to assess that the formation of joint services did not work so well [*χ*2(2) = 6.9, *p* = 0.032]. Managers aged 40 years or less thought more than managers aged over 40 years that an agreement on monitoring and evaluation worked well [*χ*2(2) = 7.9, *p* = 0.019]. Managers thought that the agreements on joint practices worked better in education than in other sectors [*χ*2(3) = 9.0, *p* = 0.030].

#### Barriers to cooperation

Both employees and managers thought that the most frequent barrier to cooperation was lack of resources. However, their perceptions differed from each other in regard to the prevalence of different barriers. Employees were more likely than managers to think that barriers occurred more frequently in work culture and attitudes, management practices and environmental factors. There was no difference between groups in regard to the lack of resources (Table 7).

Based on the employee responses, all the measured barriers to cooperation were associated with perceptions of cooperation in all sectors, excluding special health care. The cooperation was thought to function fairly or very good if there were few barriers to cooperation compared to a situation in which there were a moderate or large number of barriers (Table 4). Employees in municipalities of more than 15,000 inhabitants thought that the lack of resources was a more common barrier to cooperation than was thought by those in municipalities with under 4000 inhabitants [*χ*2(2) = 13.4, *p* = 0.001]. The lack of resources was also a more common barrier to cooperation in urban areas than rural ones [*χ*2(1) = 5.6, *p* = 0.021].

Based on responses from managers, the barriers to cooperation were associated with perceptions of cooperation particularly in mental health and substance abuse services. The cooperation was thought to function more poorly if there were a moderate or large number of barriers to cooperation compared to a situation in which there were few barriers. A lack of resources for cooperation was not related to perceptions of cooperation in any sector (Table 5). There was also no difference based on their background factors between the managers in their perceptions of barriers.

## Discussion

The study used a survey to assess employee and manager views on the functionality of cooperation in providing services for children and families in the municipalities, with consideration of both operational and strategic aspects. Employees and managers thought that cooperation worked well with primary health care and education services and from the managers’ perspective also with social welfare services. More critical perceptions were found concerning cooperation with special health care and mental health and substance abuse services. In the latter-mentioned services, cooperation often crosses several organisational boundaries, which often makes cooperation particularly challenging [3,6]. In addition, mental health and substance abuse services in particular have been organised in diverse ways in the Finnish municipalities [11,12]. Well-functioning cooperation at the operational and strategic level was related to good awareness of services and to agreed and well-functioning cooperation practices with few barriers to cooperation.

### 

#### Functionality of cooperation

Younger and highly educated employees and managers were more critical of the functioning of cooperation than older- and less-educated respondents. Those who are young and highly educated may have higher expectations and demands for the cooperation, which would be reflected in a more critical attitude. Employees working in a front-line managerial position at the operational level had more critical perceptions of the cooperation than those employees not working as managers. A front-line managers’ role in implementing and enabling cooperation is essential, because responsibility to enable the cooperation rests mainly on their shoulders. Front-line managers are often expected to handle the task of cooperation on their own with only poor support from their top managers [3]. Fully committed top-level and front-line management is important, so that professionals are able and motivated to cooperate with other organisations [3,8,20]. The task of management is to build trust and overcome territorial thinking [6].

As expected, in all sectors the cooperation was evaluated as functioning best when it involved employees working in the same sector as opposed to working with those of other sectors. Structures supporting cooperation, such as multi-disciplinary working groups and agreed practices, help to promote multi-sectoral cooperation. However, in respect of services for children and families, these have been created more frequently within sectors than between sectors or municipalities [9]. According to Ødegård [28] professionals collaborate more with professionals from their own organisation than with professionals of other services.

#### Structures supporting cooperation

Structures supporting cooperation were only to some extent associated with well-functioning cooperation. In Finland, the most common structures supporting cooperation for services for children and families are shared training sessions and projects, teams of experts and multidisciplinary work groups. It is far less common to have joint management groups or named persons responsible for cross-sectoral administrative matters [9]. According to Axelsson and Axelsson [15], the most successful forms of interorganisational collaboration in public health are stable multidisciplinary teams where the members know and trust each other, work closely together and have similar interests and goals. There is a clear pressure to move towards more strategic levels of collaboration in order to deliver more integrated children services. Too often the establishment of a collaborative structure is mistaken for the realisation of collaborative activity [2]. However, according to Katz and Hetheringtom [1], with cooperation, very different structures can work effectively.

#### Awareness of services

A good awareness of services was related to well-functioning cooperation in all sectors. However, one in five employees’ awareness was poor or rather poor. Insufficient knowledge of other's activities or available services has been found to be a barrier to cooperation in children's services [6,20,21]. In Finland, the heads of sectors are familiar with the services of other corresponding sectors, while the services of sectors in different fields and local services provided by private and voluntary organisations are less well known [9]. In any case, poor communication has been found to be a barrier to cooperation [1,2,4,18,22]. On the other hand, multi-agency working has shown to increase knowledge, understanding, and trust, as well as to improve relationships and communication between organisational units [18,19].

Cooperation was considered to function somewhat better in smaller rural municipalities than in large cities. This raises particular challenges concerning cooperation as Finnish municipal reforms aim to create larger municipalities and units for service production [11,12,14]. In small municipalities, the services of different sectors are often physically close to each other, so the implementation of cooperation may be more easily realised than in larger municipalities. Services that are physically located at a distance from each other can be a significant barrier to cooperation [21]. In addition, matching geographical and administrative boundaries between separate service sectors and authorities act to promote cooperation [7].

#### Agreed cooperation practices

When agreed cooperation practices were seen to function very well, this was associated with evaluations that the cooperation in general functioned well. A well-working information flow was also a particularly significant factor for successful cooperation. The importance of a flexible information flow as part of successful cooperation has also been identified in previous studies [3]. In Finland, there are more agreed cooperation practices within sectors than between sectors or municipalities. Practices are most frequent in the social welfare and health care sectors. On the other hand, the education sector has been most active in making cooperation agreements with the voluntary sector and private service providers [9].

#### Barriers to cooperation

Barriers in work culture and attitudes, management practices and environmental factors were associated with perceptions of poor cooperation. Corresponding barriers have also been identified in earlier studies [3,6,15]. An absence of clear leadership and a lack of support from upper management have been revealed as particularly damaging [18]. A lack of resources was also related to these perceptions among employees, but not among managers, although both groups thought the lack of resources was the most common barrier to cooperation. Insufficient resourcing, in terms of funding, staffing and time, has been identified as an important barrier to cooperation in earlier studies [1,3,4,18,22]. According to Katz and Hetheringtom [1], adequate resources and time for both formal and informal communication between practitioners from different agencies and professions are a priority for making cooperation and integration work.

#### Employees’ and managers’ perceptions

From both the perspective of operational and strategic aspects, the cooperation was challenging if you have to cross organisational boundaries. From both employees’ and managers’ viewpoints, the cooperation functioned well within their own sector and this was related to a good awareness of services and agreed cooperation practices. However, employees more than managers thought that the barriers were significant to the functioning of cooperation. On the other hand, employees were more critical than managers in regard to barriers and cooperation practices: employees thought that there were more barriers to cooperation and poorer realisation of agreed cooperation practices in municipalities.

Employees and managers have different perspectives: employees arrive at their evaluations from the operational and patient-work level, whereas managers mainly from the strategic level [5,18]. On the operational and patient-work level, issues may show themselves in different ways and particularly in different organisational units. A manager's operating environment may be more coherent than employees, and includes, for example, budgeting. Instead, employees’ operating environment is highly variable due to the diversity of needs of children and families. Practitioners have experienced that working together over services for children within complex and ever changing practice and policy contexts is extremely challenging [27]. The professionals have acknowledged the importance of multi-agency and multi-professional working in children's services in principle, but in practice it may be stressful [7] and related to increased workload [18,19].

Given the conceptual framework of integration [15] in children and family services in Finnish municipalities, cooperation is mainly voluntary cooperation or collaboration with a high degree of horizontal integration. Coordination with a high degree of vertical integration occurs less often because most of the organisations involved do not belong to a common management hierarchy. Further research is needed on interventions that promote cooperation and integration between health care, social welfare and education services, as well as with private and voluntary organisations; the use of controlled study designs is essential but challenging to implement in an organisational context. It is necessary to evaluate the efficiency and outcomes of interventions from the perspectives of children, parents, professionals, managers and policy makers [18,19].

### Strengths and limitations

The surveys were carried out in municipalities from across the whole of mainland Finland. The power analysis showed that the data were adequate in relation to the methods of analysis. For the development and testing of survey measures, a multidisciplinary expert panel, employee focus group interviews and individual interviews with managers were used; questionnaires were pre-tested. The face-validity of measures was assessed to be good, as the measures were found to be suitable for studying children and family services in municipalities. Possible processing errors were minimised by checking the data of any abnormal values before carrying out the statistical analysis.

For both data sets the response rates (37% and 33%) were relatively low, which is quite common in municipal surveys. However, in the employee data, responses were received from municipalities of different sizes, which provided for good representation (see Table 1). In regard to the manager data, the response and non-response municipalities were compared according to information provided by national municipal records so as to minimise error and bias. In respect to most of the municipalities (85%), responses were received from at least one service sector. No statistically significant differences were found based on the size of the municipality, the municipal financial situation, or the organisational structure of services for children and families.

The findings are based on data from a cross-sectional study design which precludes any interpretation of causal effects. Results are based on self-report measures that may lead to a social desirability response bias or misunderstandings concerning survey questions. However, the questionnaires were answered anonymously and municipality-specific results were not published. It is also necessary to take into account that health care and education services had a high proportion of respondents and they rated themselves quite high concerning the functionality of cooperation. So these services may have come out better than other less-represented services. Moreover, data were collected only from Finnish municipalities, and so for these reasons, any generalisation of the results beyond these samples should be done with some caution.

## Conclusions

Cooperation in providing services for children and families in Finnish municipalities works better with employees working in primary health care and education services than with those in social welfare, special health care, or mental health and substance abuse services. From both the perspective of operational and strategic aspects, cooperation works well within one's own sector, with well-functioning cooperation associated with good awareness of services and implementation of agreed cooperation practices. Implementing cooperation between sectors and beyond organisational boundaries remains challenging. However, employees more than managers thought there were more significant barriers to well-functioning cooperation. Employees are also more critical than managers concerning the occurrence of barriers and the agreed cooperation practices.

When promoting cooperation it is important to pay particular attention to the perceptions of young and highly educated employees and managers on factors related to cooperation as well as to those employees who work in a managerial position at the operational level. It is crucial that both own-sector services and services for children and families provided by other sectors in municipalities are well-known among employees and managers. Thus, service processes can be designed to be flexible and customer-oriented, while taking into account the needs of children and families.

To enhance cooperation in municipalities and services for children and families a change in management culture is needed. The focus should be on family-oriented service delivery, as well as a good availability and accessibility of services for children and families. It is necessary to pay attention to children and families’ involvement and abilities to influence social issues in services and municipalities. Well-functioning cooperation between different service providers enables a more effective allocation of resources and eliminates duplication of services, which could bring about financial savings.

Successful cooperation also requires structures that support cooperation, such as well-functioning agreed cooperation practices and an overcoming of barriers to cooperation such as a lack of resources and problems of management practices. These results can be utilised in developing customer-oriented cross-sector services for children and families.

## Figures and Tables

**Table 1.**
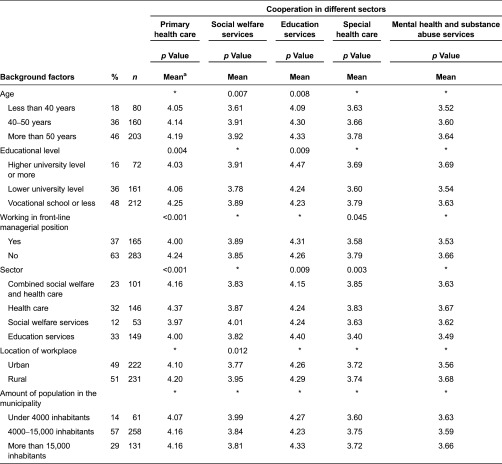
Employees’ background factors and functionality of cooperation in different sectors (*n* = 457)

^a^Independent samples *t*-test or one-way ANOVA (a paired comparison test: Scheffe). *p > 0.05.

**Table 2.**
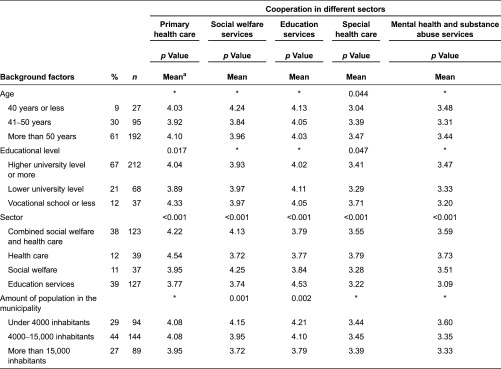
Managers’ background factors and functionality of cooperation in different sectors (*n* = 327)

^a^One-way ANOVA (a paired comparison test: Scheffe). *p > 0.05.

**Table 3.**
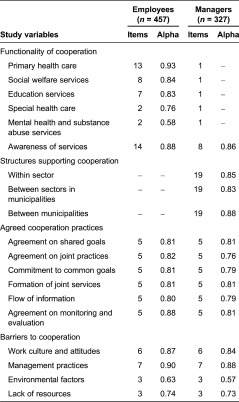
Main study variables, number of items and Cronbach's alphas

**Table 4.**
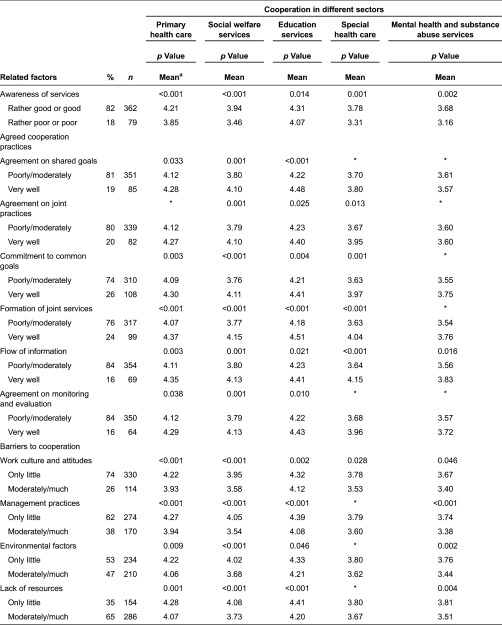
The functionality of cooperation and related factors in different sectors according to employees (*n* = 457)

^a^Independent samples *t*-test, *p* > 0.05.

**Table 5.**
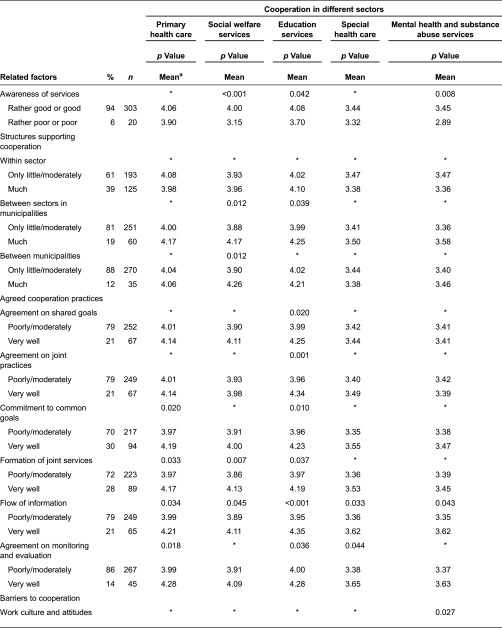

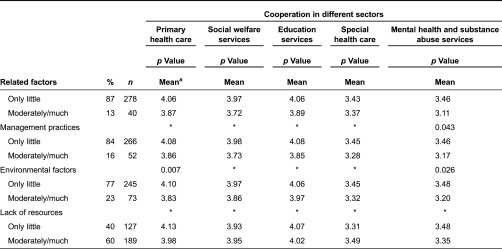
The functionality of cooperation and related factors in different sectors according to managers (*n* = 327)

^a^Independent samples *t*-test, *p > 0.05.

**Table 6.**
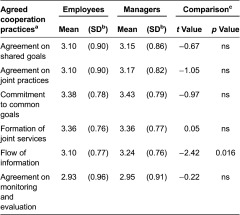
Agreed cooperation practices of services for children and families from employees’ (*n* = 457) and managers’ (*n* = 327) viewpoints.

^a^Each subscale was evaluated within sector, between sectors in municipality as well as with third-sector actors and private service providers. ^b^Standard deviation. ^c^Independent samples *t*-test.

**Table 7.**
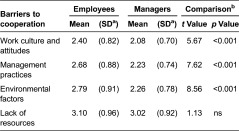
Barriers to cooperation of services for children and families from employees’ (*n* = 457) and managers’ (*n* = 327) viewpoints.

^a^Standard deviation. ^b^Independent samples *t*-test.
